# MET overexpression and activation favors invasiveness in a model of anaplastic thyroid cancer

**DOI:** 10.18632/oncotarget.26798

**Published:** 2019-03-19

**Authors:** Cyril Garcia, Camille Buffet, Laila El Khattabi, Marthe Rizk-Rabin, Karine Perlemoine, Bruno Ragazzon, Jérôme Bertherat, Françoise Cormier, Lionel Groussin

**Affiliations:** ^1^ INSERM Unité 1016, Institut Cochin, Paris, France; ^2^ Centre National de la Recherche Scientifique, Unité Mixte de Recherche 8104, Institut Cochin, Paris, France; ^3^ Université Paris Descartes, Sorbonne Paris Cité, Paris, France; ^4^ Hôpital d’Instruction des Armées BEGIN, Saint-Mandé, France; ^5^ Cytogenetics Laboratory, APHP, Cochin Hospital, Paris, France; ^6^ Department of Endocrinology, APHP, Cochin Hospital, Paris, France

**Keywords:** thyroid cancer, MET amplification, cell invasion, PHA665752, MET targeting

## Abstract

In thyroid cancers, MET receptor overexpression has been associated with higher risk of metastatic progression. In this study, it was shown that the anaplastic thyroid cancer (ATC)-derived TTA1 cell line overexpressed MET. By using FISH and relative quantification by qPCR, it was demonstrated that this overexpression resulted from a *MET* amplification with more than 20 copies. As expected, MET overexpression led to its constitutive activation and upregulated signaling towards the MAPK, PI3K/AKT, STAT3 and NF-κB pathways. Since the usual feature of *MET*-amplified cell lines is the "MET addiction" for their cell proliferation, the effect of the highly selective ATP competitive MET inhibitor PHA665752 was analyzed. While PHA665752 strongly inhibited the MAPK pathway, it did not reduce cell proliferation in TTA1 cells (IC_50_ = 4100 nM). This resistance to PHA665752 of the TTA1 cell line was demonstrated to be related to EGFR-MET functional cross-talk and PI3K/AKT and NF-κB signaling. Nevertheless, PHA665752 suppressed the anchorage-independent growth capacity of the TTA1 cell line and reduced cell migration and invasion in a transwell assay. The role of activated MET in these neoplastic properties of the TTA1 cells was also proved with si-MET-RNA targeting. Thus, this work highlights the TTA1 cell line as the first model of *MET* amplification in an ATC cell line, which leads to MET constitutive activation and underlies its neoplastic properties. Besides being a useful model for MET inhibitors screening, the TTA1 cell line also supports the argument for searching for *MET* amplification in ATC, as it could have therapeutic implications.

## INTRODUCTION

Follicular cells-derived thyroid cancers are the most frequent endocrine tumors with an increasing incidence worldwide [1, 2]. Thyroid tumors can be classified into well-differentiated (papillary and follicular thyroid carcinoma (PTC and FTC)), poorly differentiated, and undifferentiated (anaplastic) carcinoma (PDTC and ATC). PTC is the most frequent histotype accounting for 85% of thyroid cancers with a good clinical prognosis [3]. Nevertheless, about 10% of patients develop progressive disease with recurrence, radioactive iodine-resistance and distant metastasis [4]. PDTC and ATC are less common (6 % and 1-2 %), but more aggressive and associated with very poor prognosis, with a median survival of 2–10 months for ATC [5]. Progress in the identification of genetic alterations underlying the pathogenesis of thyroid carcinoma provides a basis for the development of targeted therapies for resistant and iodine-refractory cancers [6–8]. Tyrosine-kinase receptor genes rearrangements (RET/PTC, NRTK…), RAS (N-RAS, H-RAS, K-RAS) and BRAF activating mutations are frequent and mutually exclusive driver molecular abnormalities leading to a constitutive activation of the MAPK pathway in PTC [9, 10]. Other less frequent alterations, such as *PIK3CA*, *PTEN* or *CTNNB1* mutations affect the PI3K/AKT and WNT-βcatenin pathways [9]. Gene amplifications are additional genomic events in thyroid cancers, with, essentially, copy-number gains of genes encoding receptor tyrosine-kinases (RTK), such as *EGFR, PDGFRA, PDGFRB, VEFGR, KIT* and *MET* [9].

MET is the trans-membrane tyrosine kinase identified as the high affinity receptor for hepatocyte growth factor (HGF). The binding of HGF and activation of the tyrosine kinase domain provide multiple docking sites for SH2 molecules through autophosphorylation of Tyr1349 and Tyr1356. These molecules act as intracellular transducers for PI3K-AKT, RAS-MAPK and STAT3 pathways by which MET activation promotes different cellular responses, such as proliferation, cell survival, cell scattering/migration and morphogenesis [11, 12]. Deregulated HGF-MET signaling is implicated in oncogenesis and therapeutic resistance in several cancers. The migration response to MET activation contributes to the biological basis of invasion and metastasis in various neoplasms, and the cell survival response mediates drug resistance. MET is not expressed in normal thyroid cells, but its overexpression was frequently reported in thyroid carcinoma and associated with adverse outcomes [13]. Numerous studies reported the significant correlation between MET overexpression and a high risk of metastatic dissemination in PTC. However, cellular models of MET-overexpressed thyroid cancers were not yet described and the biological and therapeutic impacts of constitutively activated MET signaling were not directly investigated in thyroid cancers.

In this study, among a panel of 11 human thyroid cancer cell lines, the amplification and overexpression of the *MET* gene in the TTA1 ATC-derived cell line was described. It was postulated that MET overexpression and constitutive activation of downstream signaling pathways could have a role in neoplastic properties of this cell line. By the use of a specific pharmacological inhibitor, PHA665752, and si-RNA mediated MET downregulation, it was demonstrated that the activation of the MET-dependent signaling pathways in the TTA1 cell line contributes to neoplastic properties by sustaining anchorage-independent cell growth, cell motility and invasiveness rather than to proliferation and apoptosis protection.

## RESULTS

### MET is overexpressed and constitutively activated in the TTA1 cell line

**Figure 1:**
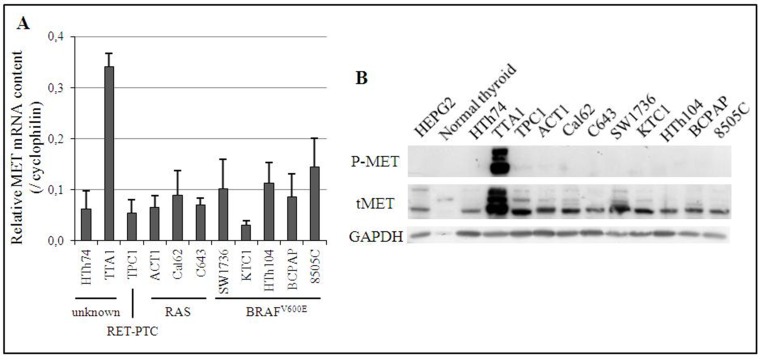
Expression of MET in 11 human thyroid cancer cell lines (**A**) Expression of MET mRNA. The relative quantification of MET mRNA was calculated by SYBR GREEN^®^ RT-qPCR with cyclophilin as the reference gene. The Cq MET/Cq cyclophilin ratio is presented. Cell lines have been classified according to their known alteration of the MAPK pathway. (**B**) Expression of MET protein. Phosphorylated and total expression of MET protein in one normal human thyroid tissue and 11 human cancer cell lines were assessed by Western blot. HEPG2 cell line is a positive control of MET protein expression.

The expression of MET mRNA was analyzed in eleven thyroid cancer cell lines, including 3 PTC cell lines (TPC1, KTC1 and BCPAP) and 8 ATC cell lines (HTh74, TTA1, ACT1, CAL62, C643, SW1736, HTh104 and 8505C). With the exception of the HTh74 and TTA1 cell lines, all of them bear an identified driver genomic alteration (RAS or BRAF activating mutation, or RET-PTC rearrangement) leading to a constitutive activation of the MAPK pathway. As shown in Figure 1A, the TTA1 cell line expressed 2.5 to 11 times more MET mRNA than the others. The TTA1 cells also exhibited overexpression of MET protein, compared to the other thyroid carcinoma-derived cells, normal human thyroid tissue and the human hepatocellular carcinoma cell line HEPG2, which served as control for MET expression (Figure 1B). The overexpression of MET in TTA1 cells was associated with a high level of constitutively activated MET receptors, as demonstrated by the high level of phosphorylation on tyrosine residues 1234/1235 (Figure 1B). And no HGF mRNA expression could be demonstrated by qRT-PCR in TTA1 cells compared to the high level of expression in the HGF-producing HL60 cell line [14] (data not shown), thus indicating that MET constitutive activation in the TTA1 cell line was not dependent on the co-expression of its ligand.

**Figure 2:**
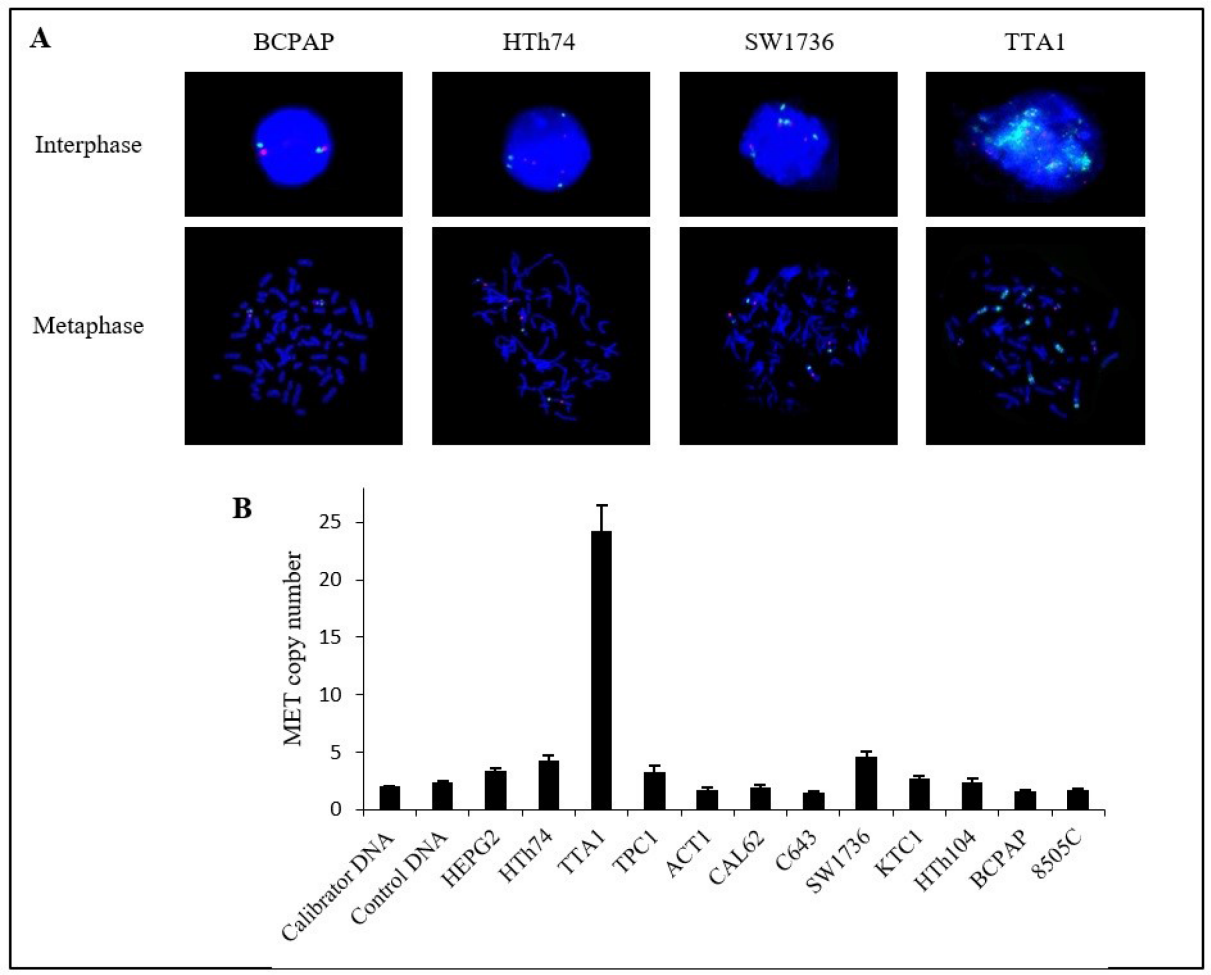
Copy number of *MET* gene in thyroid cancer cell lines (**A**) Fluorescence *in situ* hybridization in 4 thyroid cancer cell lines. *MET* probe: green; centromeric probe for chromosome 7 (*CEP7)*: red; nuclei: blue (DAPI) (magnification ×60). (**B**) Relative copy number of *MET* gene in 11 human thyroid cancer cell lines. *MET* copy number was estimated by relative quantification of the *MET* gene locus with an unrelated control locus, *COL6A5* at chromosome locus 3q22.1 by SYBR^®^Green qPCR. Calibrator DNA was leukocytes DNA from a healthy subject and control DNA was extracted from another healthy subject. A copy number >4 has been considered significant. The HEPG2 liver cancer cell line served as control.

Since MET overexpression is frequently due to *MET* amplification [15], *MET* copy number in the TTA1 cells in comparison to low MET-expressing cells was analyzed. FISH experiments demonstrated that TTA1 cells possessed a high copy number of the *MET* gene, compared to three thyroid carcinoma cell lines (BCPAP, HTh74 and SW1736), which expressed low levels of MET (Figure 2A). As determined by relative quantification of the *MET* locus, TTA1 cells possessed more than 20 copies of the *MET* gene, while the other PTC/ATC cell lines have no more than 4 *MET* gene copies (Figure 2B).

### Overexpressed MET leads to a constitutive activation of downstream signaling pathways in the TTA1 cell line

**Figure 3:**
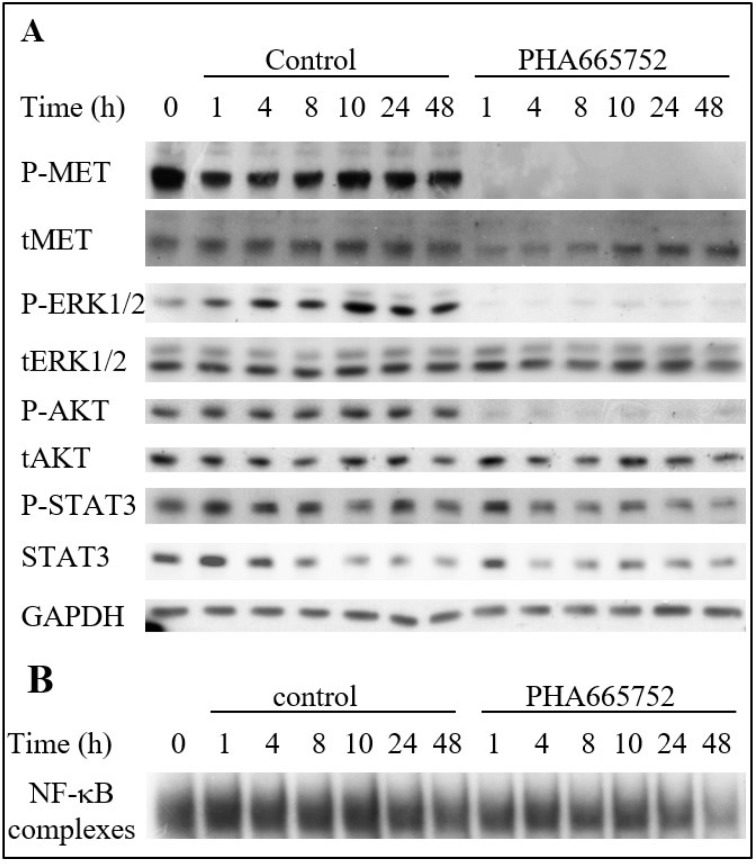
The MET inhibitor PHA665752 affects MAPK, PI3K and NF-κB pathways in the TTA1 cell line TTA1 cells were treated with PHA665752 (200 nM) or DMSO for control during the indicated times. Expression of the indicated proteins was assessed by western blot (**A**) and NF-κB activation was assessed by EMSA (**B**).

**Figure 4:**
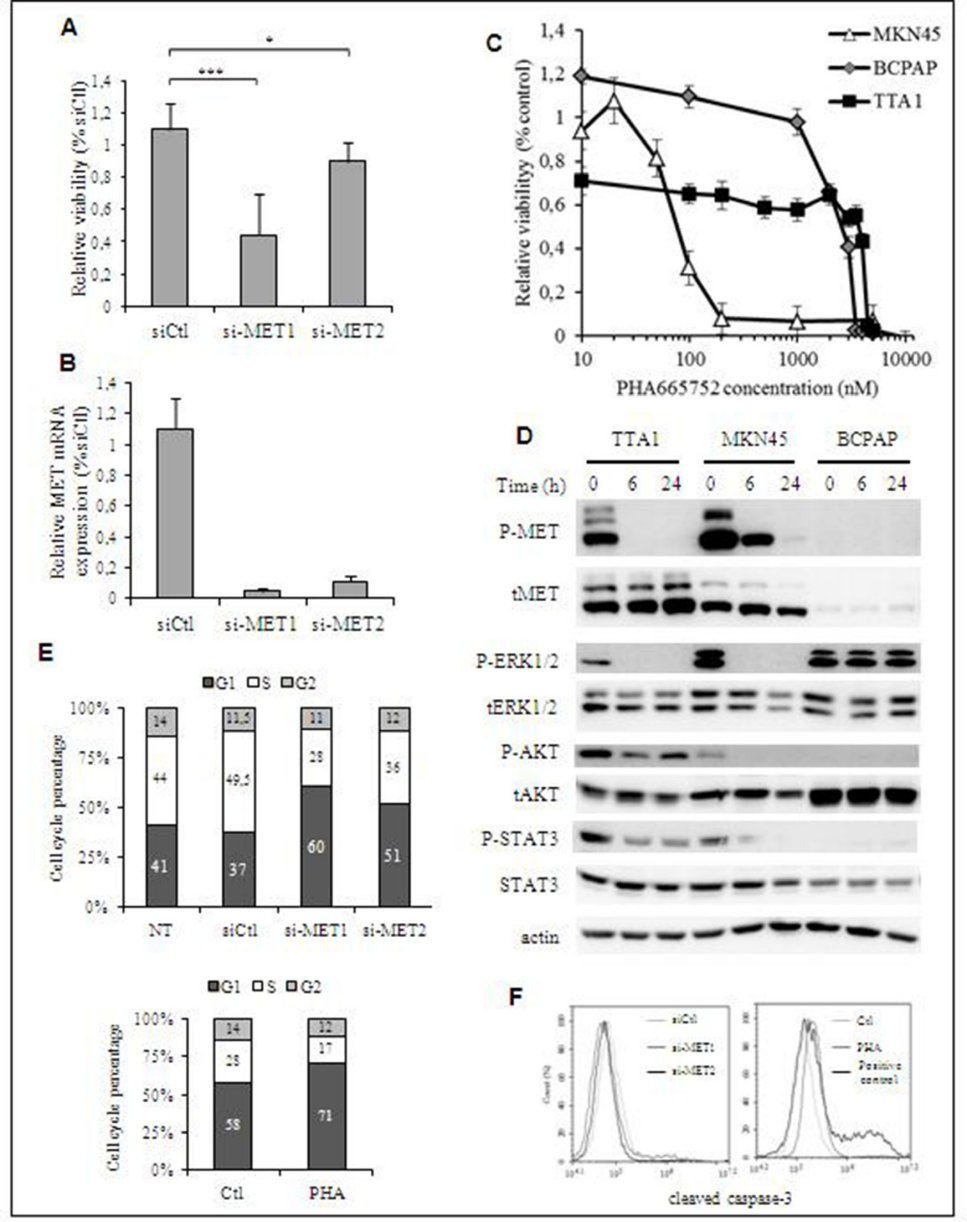
Effect of MET inactivation in TTA1 cells (**A**) TTA1 cells were transfected with two MET siRNA and one control siRNA (siCtl). Cell viability was determined at day 2 by using the crystal violet staining assay. The mean results of 8 replicates in four representative experiments are reported. (**B**) MET mRNA expression analysis by qRT-PCR in TTA1 cells 2 days after transfection with the siCtl and the 2 MET si-RNA used in cell viability assay (**C**) TTA1, BCPAP and MKN45 cells were treated with increased doses of PHA665752 over the course of 48 h. Cell viability was analyzed using the MTT assay. The MKN45 gastric cancer cells (with *MET* amplification) were used as sensitive to PHA665752 (IC_50_ = 70.8 nM). The BCPAP thyroid cancer cells (without any *MET* amplification) were used as a model of resistant cell line (IC_50_ = 2500 nM). (**D**) Western blot analysis of activated and total MET, ERK1/2, AKT and STAT3 in TTA1, BCPAP and MKN45 cells treated with 200 nM PHA665752 over a period of 6 or 24 hours. (**E**) Cell cycle analysis after propidium staining of TTA1 cells 2 days after transfection with MET si-RNAs (upper panel; NT: not transfected cells) or treated with 200 nM PHA65752 for 48 hours or not (lower panel). The mean results of two independent experiments are represented. The numbers in columns indicate the percentage of cells in G1, S and G2 cell phase, as indicated. (**F**) Apoptosis analysis by cleaved caspase-3 staining of si-MET transfected TTA1 cells (left panel) or TTA1 cells treated with 200 nM PHA65752 during 48 hours or not (right panel).

Therefore, the impact of constitutively activated MET in TTA1 cells on its downstream signaling pathways was analyzed. The highly selective ATP competitive MET inhibitor PHA665752 [16] was used to analyze the MET contribution to the MAPK, PI3K/AKT, STAT3 and NF-κB pathways activation. As shown in Figure 3A, PHA665752 induced a rapid, drastic and sustained decrease in the phospho-ERK1/2 MAPK, indicating a strong inhibition of the MAPK pathway. The AKT pathway was also inhibited but to a lesser extent according to the experiments (Figures 3A and 4D). The STAT3 pathway was affected slightly by PHA665752, as demonstrated by the partial decrease of phospho-STAT3 after 1 to 48 hours of treatment (Figure 3A). Likewise, the constitutive activation of the NF-κB pathway in TTA1 cells was partially dependent on MET activation, since PHA665752 induces a mild and late decrease of activated NF-κB complexes, as detected in gel shift experiments (Figure 3B).

### MET weakly controls cell proliferation in TTA1 cells

Thereafter, the role of MET in the TTA1 cell proliferation was analyzed by using small interfering RNA (siRNA)-mediated MET downregulation, or a pharmacological MET inactivation. As shown in the Figures 4A, 4B, the downregulation of MET expression slightly reduced cell growth for the 2 days after MET siRNA transfection, while MET mRNA expression was drastically downregulated. Advantage was then taken of the selective inhibitory effect of PHA665752 on MET activation and its downstream signaling to further evaluate its contribution to the TTA1 cell proliferation. Unexpectedly, the overactivation of MET in TTA1 cell line was not associated with a sensitivity to PHA665752 proliferative inhibition (IC_50_ = 4100 nM; Figure 4C). By comparison, the proliferation of the PHA665752-sensitive MKN45 gastric cancer cell line was inhibited with an expected IC_50_ of about 70 nM [17]. And the BCPAP thyroid cancer cell line, used as a negative control which expresses a low level of MET, displayed a weak cell proliferation inhibition with a 2500 nM IC_50_ (Figures 4C, 4D). TTA1 cells were also resistant to cabozantinib and crizotinib, two multikinase inhibitors (MKI) used in MET targeting clinical trials, while MKN45 cell growth was sensitive to these MKI, but not the BCPAP cell growth (Supplementary Figure 1). While MET activation was strongly abolished in both TTA1 and MKN45 cells, leading to the dramatic inhibition of the MAPK-ERK pathway, AKT and STAT3 activation in TTA1 cells was partially affected by the drug as compared to MKN45 cells, in which these pathways were strongly inhibited (Figure 4D). Cell cycle analysis demonstrated that MET downregulation by si-RNA, like PHA665752-mediated MET inactivation, slightly reduced cells in S phase with a slight accumulation in G1 cell cycle phase (Figure 4E). And cleaved caspase-3 staining shows that neither si-MET RNA nor PHA665752 induced apoptosis in TTA1 cells (Figure 4F).

### PHA665752 resistance of the TTA1 cell line is mediated by the EGFR, PI3K/AKT and NF-κB signaling

We then explored the molecular basis of the PHA665752 resistance of the TTA1 cells with the hypothesis that it could be mediated by the PI3K/AKT, STAT3 pathways and/or NF-κB pathways that were not totally inhibited by PHA665752-induced MET inactivation. Since functional cross-talk of MET was described in various cancers with other RTKs [11, 18], we also analyzed the eventual cross-talk of MET with EGFR which is known to be significantly expressed in the TTA1 cell line [19].

**Figure 5:**
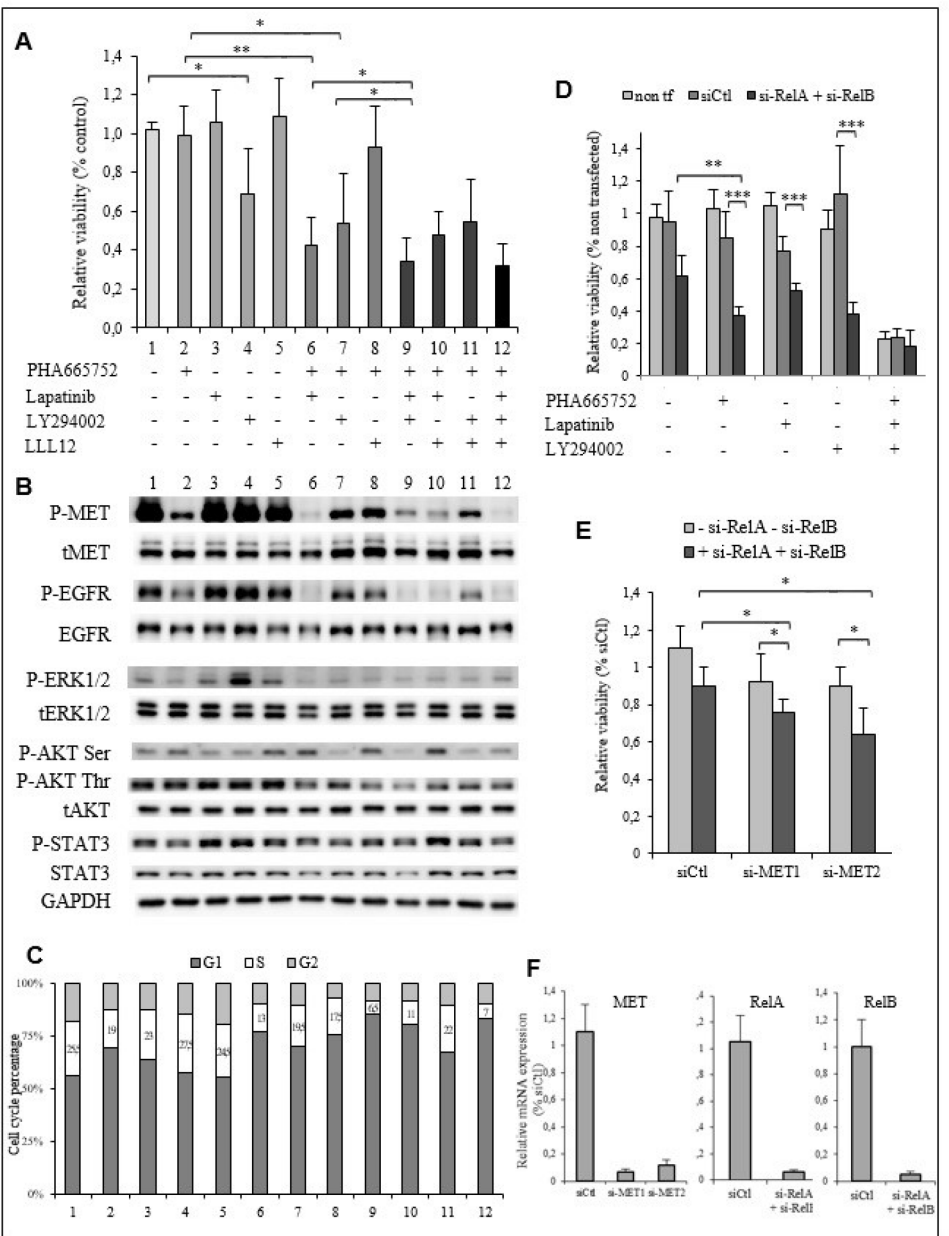
The EGFR, PI3K/AKT and NF-κB signaling pathways are implicated in PHA665752 resistance of the TTA1 cells (**A**) The TTA1 cells were incubated with the PHA665752, lapatinib, LY294002 and LLL12 inhibitors alone or in combination, as indicated. Cell viability was quantified by the crystal violet staining assay on day 3. (**B**) Western blot analysis of phosphorylated and total MET, EGFR, ERK1/2, AKT and STAT3 in TTA1 cells incubated with the combination of inhibitors used in viability assay reported in Figure 5A. (**C**) Cell cycle analysis after propidium staining of TTA1 cells incubated for 2 days with the various inhibitors combinations, as indicated in Figure 5A. (**D**) TTA1 cells were transfected with RelA si-RNA and RelB si-RNA and treated or not with PHA665752, lapatinib, and/or LY294002, as indicated, for 2 days. Cell viability was determined by using the crystal violet staining assay. The mean results of 8 replicates in two experiments are reported. (**E**) The TTA1 cells were transfected with the MET si-RNA1 and MET si-RNA2 with RelA si-RNA and RelB si-RNA or not. Cell viability was determined by using the crystal violet staining assay on day 3. The mean results of 8 replicates in two experiments are reported. (**F**) MET, RelA and ReB mRNA expression analysis by qRT-PCR in TTA1 cells 2 days after transfection with used in cell viability assay reported in Figures 5D–5E, as indicated.

Lapatinib, an inhibitor of the EGFR family members, did not affect TTA1 cells viability. The Pi3K inhibitor LY294002 alone slightly reduced TTA1 cell growth, thus indicating implication of the PI3K/AKT pathway in TTA1 cell proliferation. The STAT3 inhibitor LLL12 had no inhibitory effect. Interestingly, lapatinib synergized with PHA665752 to reduce cell growth by about half (Figure 5A, lane 6 compared to lanes 2 and 3). TTA1 cell growth was reduced more by combining PHA665752 and LY294002 (lane 7) as compared to LY264002 alone (lane 4). The simultaneous inhibition of MET (PHA665752), EGFRs (lapatinib) and PI3K (LY294002) induced the maximal cell growth reduction (Figure 5A, lane 9 compared to lanes 6 and 7). The addition of the STAT3 inhibitor LLL12 with PHA665752, lapatinib and/or LY294002 did not accentuate cell growth reduction induced by these inhibitors alone or in combination (Figure 5A, lane 12 compared to lane 9).

The consequences of these inhibitors alone or in combinations on the activation of their target and downstream signaling are reported in Figure 5B. It was observed that the combination of lapatinib with PHA665752 (lane 6) led to a more drastic inactivation of MET, as compared to PHA665752 (lane 2) alone for which a low level of P-MET persisted. In the same manner, lapatinib alone (lane 3) did not significantly reduce P-EGFR level, while no P-EGFR was detected with the combination PHA665752-lapatinib (lane 6), indicating that EGFR inactivation also requires MET inactivation. This cooperative effect between the EGFR and MET inhibitors was observed slightly on the ERK1/2 and AKT pathways with a slightly lower P-ERK1/2 and P-AKT Thr308 level. The PI3K inhibitor LY294002 (lane 4) alone reduced AKT phosphorylation only slightly, but cooperated with PHA665752 (lane 7) to induced a more pronounced decrease in AKT phosphorylation. Adding LY294002 to the PHA665752-lapatinib combination reduced (lane 9) P-AKT Ser473 and Thr308 levels more drastically. The level of P-STAT3 was slightly affected by these inhibitors combinations. We observed that the maximal cell proliferation inhibitory effect, which is achieved by the combination PHA665752-lapatinib-LY294002, was associated with the drastic inactivation of the MET and EGF receptors and the maximal inactivation of the PI3K/AKT pathway. The cell cycle analysis showed that the combination PHA665752-lapatinib-LY294002 resulted in the highest decrease of cells in the S-phase (Figure 5C).

The implication of the NF-κB pathway in the PHA resistance of the TTA1 cells was investigated by downregulating the expression of RelA and RelB, the NF-κB family members activated in the classical and alternative NF-κB pathways, respectively [20] (Figure 5D). RelA and RelB downregulation more strongly reduced cell growth in presence of PHA65752, lapatinib or LY294002, compared to the inhibitory effect of their downregulation without these inhibitors. Interestingly, the RelA and RelB downregulation did not accentuate the cell growth inhibition induced by the combination of these three inhibitors. The cooperation of si-RelA and si-RelB with the repression of MET expression by siRNA was also observed (Figure 5E). Figure 5F shows the strong inhibition of MET, RelA and RelB mRNA in these RNA targeting experiments.

Thus, these experiments demonstrated that PHA665752 resistance is related to a functional cross-talk between MET and EGFR and the activation of the PI3K/AKT and NF-κB pathways in the TTA1 cells.

### MET activation contributes to the neoplastic properties of the anaplastic TTA1 thyroid cancer cell line

We evaluated the contribution of MET activation in the neoplastic properties of this ATC cell line, such as their anchorage-independent growth capacity and their migratory and invasive potential.

**Figure 6:**
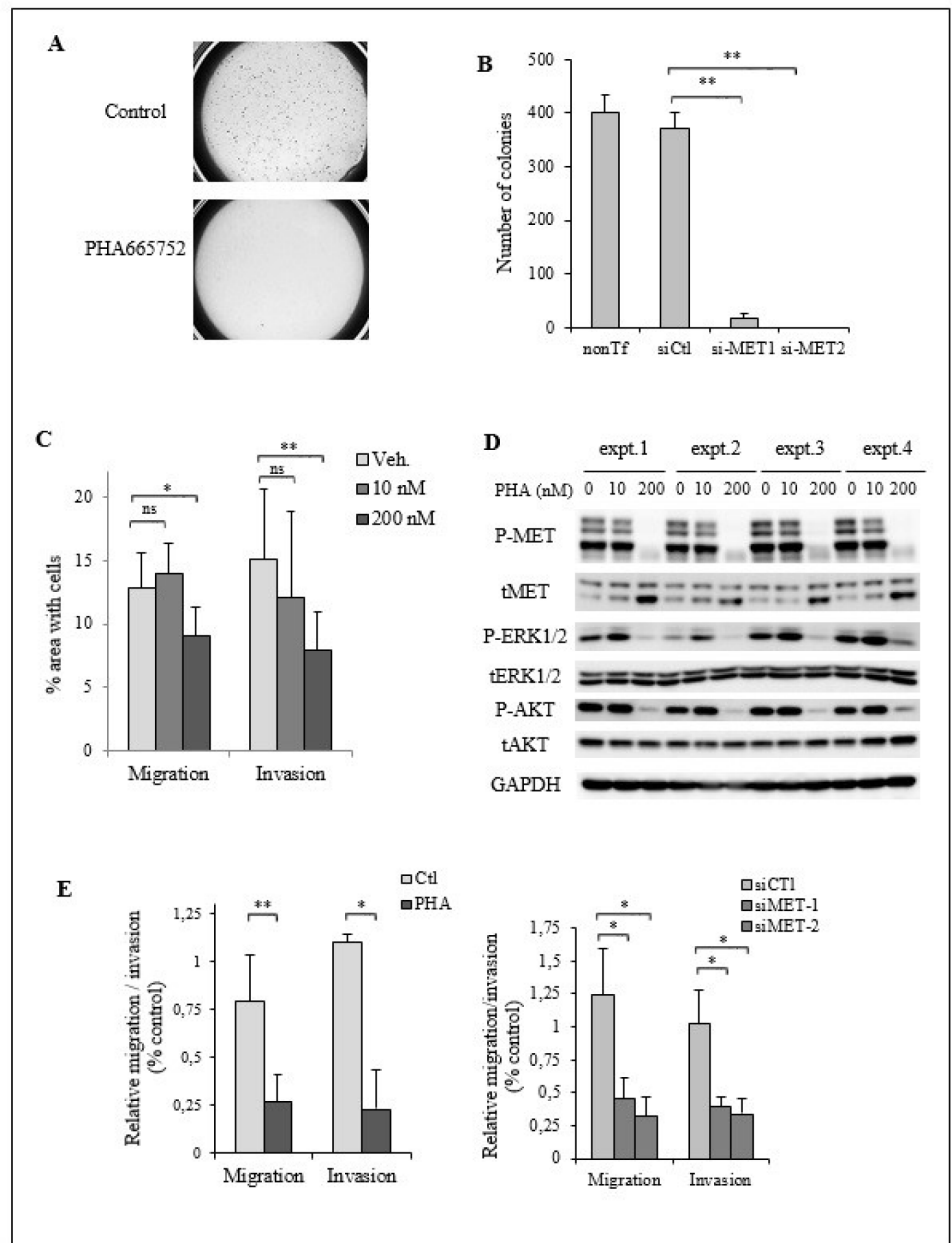
MET activation is required for cell migration and invasion in TTA1 cells (**A**) TTA1 cells were treated with PHA665752 200 nM or not for 24 hours and plated in triplicate in a soft agar medium. Low magnification image capture of one representative soft agar culture of control cells and PHA665752-treated cells are reported. (**B**) Two days after transfection with the MET siRNA1 and MET si-RNA2, TTA1 cells were plated in soft agar medium in triplicate. Colonies were counted on day 12. The mean results of triplicate of one representative experiment are reported. (**C**) Cell migration and invasion of TTA1 cells treated with the indicated doses of PHA665752 over a period of 24 h were assessed in transwell assays. The mean results of three independent migration and invasion experiments performed in duplicate are reported (*p < 0.05; **p < 0.01). (**D**) Western blot analysis of activated and total MET, ERK1/2, AKT in TTA1 cells subjected to migration and invasion assays. (**E**) Migration and invasion of TTA1 cells transfected with MET si-RNA1 and MET si-RNA2 (right panel), compared to cell migration and invasion of PHA665752-treated cells (left panel). The mean results of two independent migration and invasion experiments carried out in duplicate are reported (*p < 0.05; **p < 0.01)”.

First, the impact of MET inactivation by the PHA665752 or by siRNA-mediated downregulation was analyzed on the capacity of TTA1 cells to develop colonies in semi-solid culture. Figure 6A illustrates the strong inhibitory effect of PHA665752 on the TTA1 cell growth in soft agar cultures. The si-RNA mediated MET downregulation also drastically affected the growth capacity of the TTA1 cells in the soft agar assay (Figure 6B).

The impact of MET inactivation by PHA665752 on the migratory and invasive capacities of TTA1 cells was also analyzed. As shown in Figure 6C, cell migration and invasion of TTA1 cells in transwell assay were reduced by 200 nM PHA665752, while the MAPK and PI3K/AKT pathways were still downregulated (Figure 6D). It was observed that 200 nM was the minimal dose inducing the complete growth inhibition of the PHA665752-sensitive MKN45 cells (Figure 4C). The migratory and invasive properties of TTA1 cells was similarly inhibited by si-MET RNA compared to PHA665752 (Figure 6E), thus demonstrating that MET expression is required for cell migration and invasion capacities of the TTA1 cells.

## DISCUSSION

The tight regulation of the HGF-MET signaling that occurs in normal tissues is lost through multiple ways in several cancers. Missense activating point mutations of *MET* were reported in papillary renal cell carcinoma, hepatocellular carcinoma, small-cell lung cancer and other cancers [21]. Most of them flank the critical tyrosine residues Y1234 and Y1235 within the kinase domain and lead to the constitutive MET kinase activity with the potential to drive tumorigenesis. Several other mutations were also reported in the MET juxtamembrane and Sema domains with potential functional consequences related to MET degradation and ligand binding and dimerization, respectively. The missense T1010I mutation in the exon 14 encoding the juxtamembrane domain was first described as associated with an increased invasiveness in lung cancers [22]. In follicular cells-derived thyroid cancers, this mutation was found in 6% of the 53 PTC, 10% of the 21 FTC, but none of the 17 ATC studied [23]. Another study reported this mutation in one ATC among 34 PDTC and 18 ATC [24]. However, the functional consequences of the T1010I mutation are still unclear, since the thyroid cell line ML1 bearing this mutation was refractory to growth inhibition by the MET inhibitor SU11274. The recent deep-sequencing analysis of 8 mutational hotspots associated with aggressive progression showed that *MET* exon 2 and 14 were frequently mutated in PTC [25]. Interestingly, 28 *MET* non-synonymous point mutations were found in PTC with distant metastasis (DM-PTC), compared to only 3 in control PTC without distant metastasis. In the 39 PTC with distant metastasis, 36% harboured *MET* mutation, as opposed to 20% of the 20 control PTC, thus indicating a strong association of *MET* exon 2 or 14 mutations with tumor progression. Nevertheless, only one MET mutation was detected in the recent large scale genomic analysis of 507 non aggressive PTC [10]. In aggressive thyroid cancers, one mutation in Sema domain was reported in a cohort of 144 ATC [26], and no mutations were detected among 84 PDTC and 33 ATC cases [27]. Gene fusion resulting in kinases activation is a common relevant mechanism in cancer malignancy and many known fusions implicating *MET* were described in different types of cancers. In thyroid cancer the first description was the *TFG-MET* fusion, found in one papillary thyroid cancer after the depth analysis of the Cancer Genome Atlas (TCGA) RNA-seq data [28].

The MET pathway oncogenic activation can also be achieved by the MET overexpression through a HGF-independent dimerization. In PTC, increased transcription of the *MET* gene was demonstrated as resulting from the upregulation of hypoxia inducible factor (HIF-1), probably through hypoxia [29]. The under-expression of miR-199a-3p in PTC specimens and cell lines was also implicated in the high level of MET expression in PTC [30]. *MET* gene amplification is the most frequent genetic alteration that leads to MET activation in non-small cell lung cancer, colorectal and gastric cancers with poor prognosis [15]. In aggressive thyroid carcinomas, frequent gains in chromosome 7 were reported (10 out of 84 PDTC and 14 out of 33 ATC) [27]. *MET* amplification has been found to be significant in a series of ATC (5 out of 42 anaplastic carcinomas (11.9%) with 3.5 to 8.7 copy number gains) [31]. Our study now provides the first demonstration of *MET* amplification in an ATC-derived cell line with a biological impact related to neoplastic properties.

In order to know whether *MET* amplification contributes to the tumoral properties of the TTA1 cells, an RNA interference-based strategy and a pharmacological approach with PHA665752, a MET inhibitor with a high selectivity (Ki 4 nM) for the catalytic domain of the kinase, were used [16]. It is generally accepted that *MET*-amplified cancer cells are highly sensitive to MET inhibitors [32, 33]. In gastric cancer cell lines with high *MET* amplification (fold>8), 50 nM of PHA665752 were enough to inhibit cell growth [34]. In other studies, *MET*-amplified cell lines were sensitive to 200 nM PHA665752 or presented an IC_50_ between 20 to 100 nM, while cell lines without *MET* amplification were resistant to the drug [17, 33]. The high sensitivity of cancer cells with *MET* amplification defines their “MET addiction”, which is related to the constitutive activation of this receptor, because of its ligand-independent dimerization when overexpressed at the cell surface. In the TTA1 cells, it was demonstrated that MET overexpression was also associated with its constitutive activation leading to the stimulation of downstream signaling pathways. However, the high level of amplification with more than 20 copies of *MET* was not associated with a high sensitivity of TTA1 cells to MET inhibition by PHA665752. A lack of interaction between PHA665752 and its target could not be asserted, since the drug undoubtedly produced its expected inhibitory effects on MET receptor activation and the downstream signaling effectors ERK1/2 and AKT.

In cancers, MET activation is commonly implicated in both cell proliferation and invasiveness [12]. However, our study demonstrated the dissociation between inhibition of the TTA1 cell line proliferation and inhibition of its motility and invasiveness by the PHA665752 MET inhibitor. The TTA1 cell growth unsensitivity to MET pharmacological inactivation was further demonstrated with cabozantinib and crizotinib, despite the lower specificity of these multikinase inhibitors toward MET. The dissociation of the mitogenic activity from the pro-invasive activity of MET was already demonstrated by the differential response of the human breast cell line 184B5 to the naturally occurring truncated HGF isoform, HGF/NK2 [35]. Indeed, HGF/NK2 induced cell motility, but not DNA synthesis, while MET and the downstream MAPK pathway were activated. This study thus indicated that MET activation can occur without signaling toward a mitogenic response. The uveal melanoma recently gave another model in which MET is required for cell migration and invasion, and not proliferation [36]. The cell growth of uveal melanoma cell lines was described as not being sensitive to the MET inhibitor crizotinib and MET downregulation, while crizotinib prevents their metastasis potential.

Then, we used the TTA1 cells to investigate the molecular basis of the resistance to MET inhibitors, which frequently occurs with MET targeting therapies. We demonstrated that TTA1 cell proliferation is dependent on the cooperation between MET and EGFR, which act in cross-talk to activate downstream signaling pathways, such as the PI3K/AKT and NF-κB pathways. These are two pathways that we demonstrated as being necessary for TTA1 cell proliferation. Nevertheless, the hypothesis that other RTKs-mediated PI3K and NF-κB activation could also drive cell proliferation in the TTA1 cells cannot be excluded. Functional cross-talk of MET with other RTKs, such as EGFR, HER2, HER3, RET or IGF1R, was identified in various cancers as mediating inhibitors resistance [18, 37–40]. Our work now gives evidence for considering the TTA1 cell line as a new model of MET inhibitors resistance by a functional cross-talk between MET and EGFR. And the NF-κB pathway activation which also participates in the PHA665752 resistance of TTA1 cells emerged as a molecular mechanism implicated in the development and the therapeutic resistance of thyroid cancers [41].

In the TTA1 cells, it was further demonstrated that MET activation contributes to their neoplastic properties such as the anchorage-independent growth and migration and invasion capacities, thus corroborating the numerous histological analyses demonstrating the significant correlation between MET overexpression and a higher risk of metastasis in thyroid cancers [42, 43]. In PTC, MET overexpression was associated with high responsiveness to HGF, thus leading to increased invasiveness and Cox-2-dependent release of chemokines and angiogenic factors [44]. The MET overexpression together with its constitutive activation enhanced the migration and invasion of cells of the TPC1 PTC cell line, while its invalidation negatively affected the HGF-induced cell migration [45]. MET activation was also implicated in the acquired resistance to the BRAF inhibitor PLX4032 (vemurafenib) in an ATC cell line by mediating PI3K/AKT activation, apoptosis protection and epithelio-mesenchymal transition [46, 47]. More recently, *MET* amplification was demonstrated as a central mechanism to BRAF inhibition resistance in a murine model of ATC [48]. In other PTC cell lines, high doses (> 1 µM) of the MET inhibitor PHA665752 were necessary to reduce their cell growth and migration and invasion capacities [49, 50]. Therefore, the TTA1 cell line is the first ATC cellular model of a MET-dependent migration/invasion highly sensitive to the MET inhibitor PHA665752, and because of that it constitutes a powerful tool for MET inhibitors study.

Several MET targeting-MKI and specific MET kinase inhibitors have entered clinical trials in various cancers with MET alterations [51]. In this field, two MKIs were assessed for treatment of advanced and radioiodine refractory thyroid cancers. Crizotinib, which targets both Anaplastic Lymphoma Kinase (ALK) and MET, has been reported with a remarkable response in a woman with *ALK*-rearranged ATC [52], and entered an ongoing phase II study for the treatment of solid tumors including MET-mutated thyroid cancers (https://clinicaltrials.gov/ct2/show/NCT02034981). Cabozantinib (XL184), targeting VEGFR1/2, MET, RET, KIT and FLT3, has been assessed in several cell line models and has been approved by the FDA for the treatment of progressive medullary thyroid cancer [53]. In a phase I study with cabozantinib in metastatic differentiated thyroid cancer, a partial response was reported in 8 out of 15 patients who failed standard radioactive iodine therapy and in 5 out of 8 patients previously treated with VEGF pathway inhibitors [54]. A phase II study recently confirmed the clinical benefit of cabozantinib in patients refractory to radiotherapy and non-responsive to a VEGF- targeted therapy [55]. A phase II study with cabozantinib in radioiodine-refractory differentiated thyroid carcinoma in first line setting is currently ongoing (https://clinicaltrials.gov/ct2/show/NCT02041260). Analyses of the genetic alterations in follicular thyroid cancers were essentially focused on mutations with sequencing analysis, and gene amplifications were not extensively investigated. This work argues for the importance of further investigation into *MET* amplification in these cancers, as it could have therapeutic implications for patients who are refractory to standard therapy.

## MATERIALS AND METHODS

### Cell lines

The human hepatocellular carcinoma cell line HEPG2 was kindly provided by C. Perret (Cochin Institute, Paris, France). The human anaplastic thyroid cancer cell lines HTh74, TTA1, ACT1, CAL62, C643, SW1736, HTh104 and the human thyroid papillary cancer cell lines TPC1 and KTC1 were kindly provided by J. Fagin (Memorial Sloan Kettering Cancer Centre, New York, USA). These cell lines have been previously authenticated [56]. The human thyroid cancer cell lines BCPAP and 8505C and the human gastric cancer cell line MKN45 were purchased from the German Collection of Microorganisms and Cell Culture (Braunschweig, Germany). The human promyelocytic cell line HL60 was used as a positive control for HGF expression. Molecular characteristics of cell lines are listed in Supplementary Table 1. The cell lines were cultured either in RPMI or in DMEM medium supplemented with 5–10% fetal calf serum (Life Technologies) and antibiotics (Life Technologies).

### Reagents

The selective ATP competitive MET inhibitor PHA665752 was purchased from Sigma-Aldrich, and the EGFRs inhibitor lapatinib, the PI3K inhibitor LY294002 and the STAT3 inhibitor LLL12 were purchased from Selleckchem. The cabozantinib and crizotinib RTKs inhibitors were kindly provided by B. Blanchet (Cochin Hospital, Paris). The inhibitors were diluted in dimethylsulfoxide (DMSO).

The following antibodies were from Santa Cruz Biotechnology, Inc. (Santa Cruz, CA): anti-MET (sc-10), anti-P-ERK1/2 (sc-7383), and anti-GAPDH. The anti-P-MET (Tyr1234/1235)(D26), anti-ERK 1/2, anti-P-AKT (Ser473), anti-P-AKT (Thr308), anti-AKT, anti-P-STAT3 (D3A7), anti-STAT3, anti-P-EGFR (Tyr1068), anti-EGFR antibodies and anti-cleaved caspase-3 (Asp15) were from Cell Signaling Technology, and the anti-actin antibody was from Sigma-Aldrich.

### Western blotting

Total cell protein lysates were extracted using Tris-NP40 buffer (20 mM TrisHCl (pH 7.5) 1 mM EDTA, 150 mM NaCl, NP40 1%) containing protease and phosphatase inhibitors (Roche). Protein concentration was determined using the Bradford reagent (BioRad). Proteins were separated by 7–12% SDS-PAGE and transferred on nitrocellulose membranes (GE Healthcare). After blocking with 5% non-fat milk or 5% bovine serum albumin, proteins were immunoblotted overnight with primary antibodies followed by incubation with HRP-conjugated secondary antibodies. Immunostaining was revealed by chemiluminescence (Pierce™ ECL, Thermo Scientific) and visualized using the ImageQuant LAS4000 imaging system (GE Healthcare).

### Electrophoretic mobility shift assay (EMSA)

Electrophoretic mobility shift assay (EMSA) was carried for analysis of NF-κB activation, as previously described [57]. Briefly, total cell extracts prepared in a high salt buffer were analyzed using the radiolabeled HIV-LTR tandem κB oligonucleotide as κB probe [58].

### Cell RNA extraction and qRT-PCR

RNA extraction was performed with RNeasy™ Mini Kit (Qiagen) according to manufacturer’s instructions. RNA were retrotranscribed in cDNA by using the high capacity cDNA reverse transcription kit (Applied Biosystems™, ThermoFisher). Quantification of mRNA was performed by RT-qPCR SYBR Green in capillaries (Light Cycler Carousel-Based system, Roche, France) or in 96-microwell plates (Light Cycler 480, Roche). Values of mRNA were calculated by relative quantification using the 2^-∆∆CT^ method, with *PPIA* as the reference gene. Oligonucleotide sequences are detailed in Supplementary Table 2.

### Genomic DNA extraction and qPCR (quantitative polymerase chain reaction)

DNA was extracted with QIAGEN™ QIAamp™ DNA Mini Kit and QIAamp Mini spin columns according to manufacturer’s instruction. Nucleic acids quantification was made by spectrophotometry (Nanodrop 2000, Labtech). Cell line DNA quantification was performed by real time PCR using SYBR^®^Green probes in 96-microwell plates in Light Cycler 480 (Roche). Oligonucleotide sequences are detailed in Supplementary Table 3.

Relative quantification of *MET* copy number was calculated using *COL6A5* as the reference gene, located at 3q22.1. This gene has been chosen after verification of the stability of the ratio *ALB/COL6A5* for all cell lines. The results of relative quantification was normalized with normal healthy human DNA containing 2 copies of the *MET* gene, one sample serving as calibrator, the other serving as control.

### Fluorescence *in situ* hybridization (FISH)

FISH analysis was performed on metaphase spreads obtained according to standard protocols using Zyto*Light*™ SPEC MET/CEN7 Dual color probe (Clinical Sciences). The control probe with orange dye targets the centromere of chromosome 7 (D7Z1), serves to locate the chromosome of interest and the specific probe with green dye targets area genomic locus including the *MET* gene located at 7q31.2 (D7S486-D7S2347). Six metaphases and 6 nuclei were analyzed for each sample.

### Cell viability assay

Cells were seeded in 96-microwell plates and viability was assessed by using either the MTT (Thiazollyl Blue Tetrazolium Bromide) assay, as described by Buffet and coll. [59], or the crystal violet staining assay [60]. Within each experiment, the means of 8 or 10 replicates and the standard errors of the means were calculated. IC_50_ values were estimated by non-linear logistic regression model by using the Graphprism software.

### Cell cycle and apoptosis analysis

Cell cycle was analyzed by flow cytometry as previously described [61]. Apoptosis was analysis by flow cytometry after cleaved caspase-3 staining. Cells were fixed with 4% paraformaldehyde, treated with NH4Cl and permeabilized in 0.5% NP40 in PBS. After incubation in 5% bovine serum albumin, cells were incubated with anti-cleaved caspase-3 antibody and secondary Alexa 488-coupled anti-rabbit antibody (Molecular Probes). Flow cytometry analysis was performed with the Novocyte cytometer (AREA Biosciences) by using the Novoexpress software.

### Gene silencing by RNA interference

Small interfering (si) RNAs for c-MET (siMET-1: 5′-ACAAGAUCGUCAACAAAAA-3′; siMET-2: 5′-CUACAGAAAUGGUUUCAAA-3′), RelA (si-RelA:5′- GGAUUGAGGAGAAACGUAA-3′) and RelB (si-RelB: 5′-GACTGCACCGACGGCATCT-3′) were purchased from Eurofins Genomics. Cells were transfected with Lipofectamine 2000 or Lipofectamine RNAiMAX (Life Technologies) according to the manufacturer’s protocol. Cells were either seeded in 96-well plates for cell viability assay or in 6-well plates for harvesting at day 2 for qRT-PCR analysis.

### Anchorage-independent growth assay

The anchorage independent growth was analysed through a soft agar colony formation assay, as described previously [59]. Colonies were counted after 2 weeks in culture. Low magnification images were captured on a macroscope (0.5 × objective, AZ100M, Nikon) with NIS software (Nikon).

### Cell migration and invasion assays

Cell migration and invasion were assessed in Transwell polycarbonate membrane inserts (Corning) as previously described [59]. For the invasion assay, the transwell membranes were coated with Growth Factor Reduced Matrigel (12.5 μg in 60 μl /well (BD Biosciences). After overnight incubation at 37° C, cells that had migrated or invaded the underside of the membrane were stained with crystal violet. The total membrane was scanned and analyzed using ImageJ software.

### Statistical analysis

Statistical significance was assessed using the Student’s *t*-test (GraphPad software on line). *P* values < 0.05 were considered significant with the following degrees: *p < 0.05; **p < 0.01, ***p < 0.001.

## SUPPLEMENTARY MATERIALS FIGURE AND TABLES


