# Supplementary material for: MET overexpression and activation favors invasiveness in a model of anaplastic thyroid cancer
Source: Oncotarget. 2019 Mar 19;10(23):2320–34. doi: 10.18632/oncotarget.26798 (PMC6481343; doi:10.18632/oncotarget.26798)
Supplement: Supplementary file 1 [file oncotarget-10-2320-s001.pdf]

MET overexpression and activation favors invasiveness in a model of anaplastic thyroid cancer

SUPPLEMENTARY MATERIALS

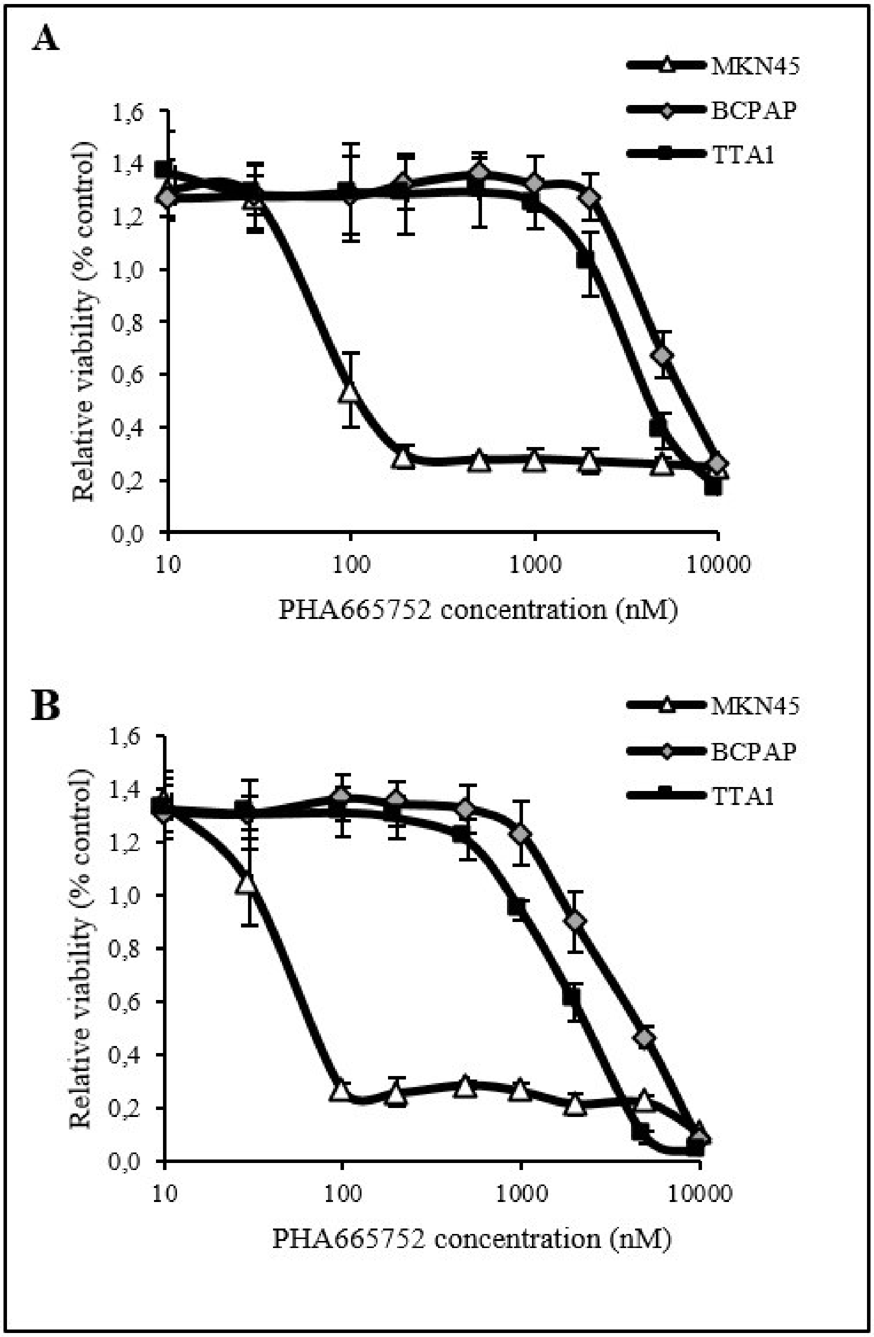

Supplementary Figure 1: The TTA1 cell line is not sensitive to the cabozantinib and crizotinib multikinase inhibitors. TTA1, BCPAP and MKN45 cells were treated with increased doses of cabozantinib (**A**) or crizotinib (**B**) over the course of 48 h. Cell viability was analyzed using the crystal violet assay. The mean results of 8 replicates in one representative experiment are reported.

Supplementary Table 1: Human cell lines characteristics

Cell lines	Type	MAPK pathway alteration
HEPG2	Hepatocellular carcinoma	No known mutation
HtH74	ATC	No known mutation
TTA1	ATC	No known mutation
TPC1	PTC	RET/PTC-1 rearrangement
ACT1	ATC	NRAS mutation
CAL62	ATC	KRAS G12R mutation
C643	ATC	HRAS G12A mutation
SW1736	ATC	BRAF V600E mutation
KTC1	PTC	BRAF V600E mutation
HtH104	ATC	BRAF V600E mutation
BCPAP	PTC	BRAF V600E mutation
8505C	ATC	BRAF V600E mutation
MKN45	Gastric cancer	No known mutation

ATC : Anaplastic Thyroid Cancer. PTC : Papillary Thyroid Cancer.

Supplementary Table 2: Primer sequences used for qRT-PCR

Gene	Primer sequence	Lenght (bp)
MET	F: 5′-ACCTGCCAGCGACATGTCTT-3′<br>R: 5′-GACACTGGCTGGGCTCTTCTATC-3′	100
HGF	F: 5′-GTAAATGGGATTCCAACACGAACAA-3′<br>R: 5′-TGTCGTGCAGTAAGAACCCAACTC-3′	116
RelA	F: 5′-TTGAGCCCACAAAGCCTTATCAAGT-3′<br>R: 5′-GGACAATGCCAGTGCCATACAG-3′	104
RelB	F: 5′-CTCACTCTCGCTCGCCGTTTC-3′<br>R: 5′-CACAGGGCCCAGGGTGACCGT-3′	172
PPIA	F: 5′-ATGGCACTGGTGGCAAGTCC-3′<br>R: 5′- TTGCCATTCCTGGACCCAAA-3′	242

PPIA : Peptidylprolyl Isomérase A (cyclophilin A).

Supplementary Table 3: **Primer sequences used for PCR**

Gene	Primer sequence	Lenght (bp)
MET	F: 5′- GGGCTATGTCCCATTTCTCA -3′<br>R: 5′- GGGCTATGTCCCATTTCTCA -3′	104
ALB	F: 5′- GGCAAAGGATGTCTTCCTGGGCAT -3′<br>R: 5′ TGGGCAGCAGGGAAATTGTAGCA -3′	129
COL6A5	F: 5′- ACCACTGGCAGCTTCTTGCAA -3′<br>R: 5′- CGCCCCTGGACATCCTGCAA -3′	121

ALB: albumin, *COL6A5*: α5 chain collagen 6.
